# Analysis of the Early Immune Response to Infection by Infectious Bursal Disease Virus in Chickens Differing in Their Resistance to the Disease

**DOI:** 10.1128/JVI.02828-14

**Published:** 2014-12-10

**Authors:** Jacqueline Smith, Jean-Remy Sadeyen, Colin Butter, Pete Kaiser, David W. Burt

**Affiliations:** a The Roslin Institute and R(D)SVS, University of Edinburgh, Easter Bush, Midlothian, United Kingdom; b The Pirbright Institute, Compton Laboratory, Compton, Berkshire, United Kingdom

## Abstract

Chicken whole-genome gene expression arrays were used to analyze the host response to infection by infectious bursal disease virus (IBDV). Spleen and bursal tissue were examined from control and infected birds at 2, 3, and 4 days postinfection from two lines that differ in their resistance to IBDV infection. The host response was evaluated over this period, and differences between susceptible and resistant chicken lines were examined. Antiviral genes, including *IFNA*, *IFNG*, *MX1*, *IFITM1*, *IFITM3*, and *IFITM5*, were upregulated in response to infection. Evaluation of this gene expression data allowed us to predict several genes as candidates for involvement in resistance to IBDV.

**IMPORTANCE** Infectious bursal disease (IBD) is of economic importance to the poultry industry and thus is also important for food security. Vaccines are available, but field strains of the virus are of increasing virulence. There is thus an urgent need to explore new control solutions, one of which would be to breed birds with greater resistance to IBD. This goal is perhaps uniquely achievable with poultry, of all farm animal species, since the genetics of 85% of the 60 billion chickens produced worldwide each year is under the control of essentially two breeding companies. In a comprehensive study, we attempt here to identify global transcriptomic differences in the target organ of the virus between chicken lines that differ in resistance and to predict candidate resistance genes.

## INTRODUCTION

Infectious bursal disease virus (IBDV) is a highly contagious virus with a bisegmented double-stranded RNA (dsRNA) genome (belonging to the family Birnaviridae) which causes immunosuppression in chickens (1). Segment A contains two overlapping open reading frames, the larger of which encodes viral proteins VP2, VP3 (both structural capsid proteins), and VP4 (a viral protease). The smaller open reading frame encodes the nonstructural protein VP5. Segment B encodes VP1 which is a multifunctional polymerase. There are two known serotypes: serotype I viruses cause a range of disease severity in chickens and are further classified into classic, variant, and highly virulent strains, and serotype II viruses are nonpathogenic. Although largely controlled by vaccination, new virulent strains of the virus mean that infectious bursal disease (IBD; also known as “Gumboro” disease) still remain a threat to the poultry industry. The virus infects dividing IgM^+^ B lymphocytes and the main site of viral replication is the bursa of Fabricius, where B cells are produced (2, 3). IBDV can also infect macrophages (3–5).

Infection is spread orally via contaminated feed and water (6). IBDV affects young birds, with the disease usually being diagnosed in 3- to 6-week-old birds. Younger birds do not show clinical signs but are immunosuppressed (7, 8). Symptoms include anorexia, depression, diarrhea, ruffled feathers, immunosuppression, and bursal lesions. Death is often due to dehydration, which leads to kidney lesions (9). The disease peaks between 2 to 5 days postinfection (dpi) and is practically cleared by day 7 (10). During this acute phase, bursal follicles are depleted of B cells, and the bursa becomes atrophic. Abundant viral antigen can be detected in the bursal follicles and other lymphoid organs such as the cecal tonsils and spleen. CD4^+^ and CD8^+^ T cells accumulate around the site of virus replication (6). Mortality is variable and tends to affect layers more than broilers but can be up to 100% with very virulent strains of the disease. Even if birds survive, the resulting immunosuppression and effect on egg production in layer birds is significant (11).

Although disease is currently controlled by vaccination, alternative control measures are being explored, including breeding for enhanced genetic resistance. Involvement of the major histocompatibility complex (MHC) in resistance to IBDV has been a topic for debate, but it does appear to have a role (12). Two specific-pathogen-free lines of chickens held at The Pirbright Institute (previously known as the Institute for Animal Health), a Brown Leghorn line and the White Leghorn line 6_1_, were previously shown to be susceptible and resistant, respectively, to IBD (13). Differences in IBDV viral loads, measured by quantitative reverse transcription-PCR (qRT-PCR), in the bursae of infected birds of these two lines were evident from as early as 1 dpi (14). In the present study, we carry out a comprehensive gene expression study, comparing the response to infection in the two lines at 2, 3, and 4 dpi, with the hypothesis that genes underlying at least some of the resistance mechanisms will be involved at this early, innate stage of the immune response.

## MATERIALS AND METHODS

### Ethics statement.

All animal work was conducted according to United Kingdom Home Office guidelines.

### Experimental animals.

Three-week-old birds from each line (6_1_ and Brown Leghorn [Brl]) were separated into two experimental rooms, with ad libitum access to food and water. In one room, 54 birds were mock infected intranasally with 100 μl of phosphate-buffered saline (PBS; 27 birds from each line). In the other room, 54 birds were infected intranasally (27 birds from each line) with 10^1.3^ 50% embryonic infectious doses (EID_50_) of the classical virulent IBDV strain F52/70 in 100 μl of PBS. The birds were monitored for clinical signs for up to 4 dpi and killed at 2, 3, or 4 dpi (nine individuals at each time point). Spleens and bursae were collected from all birds for qRT-PCR analysis for virus and host genes, for microarray analysis, and for bursal damage scores by immunohistochemistry. Blood samples were also taken from each bird for DNA isolation.

### Bursal damage scoring.

Segments of bursal tissue were fixed in formaldehyde-saline (pH 7.6) before routine histological processing and staining with hematoxylin and eosin. The degree of bursal damage was assessed using the histological scoring system (scored in the range 0 to 5, with 5 indicating the greatest level of damage) described by Muskett et al. (15), with each section being scored blindly by two separate individuals.

### RNA preparation.

Tissue samples (∼30 mg) were stabilized in RNAlater (Ambion/Life Technologies, Paisley, United Kingdom) and disrupted using a bead mill (Retsch MM 300; Retsch, Haan, Germany) at 20 Hz for 4 min. Total RNA was prepared using an RNeasy kit (Qiagen, Crawley, United Kingdom) extraction method according to the manufacturer's protocol. Samples were resuspended in a final volume of 50 μl of RNase-free water. Concentrations of the samples were calculated by measuring the optical density at 260 nm (OD_260_) and the OD_280_ on a spectrophotometer (NanoDrop; Thermo Scientific, Paisley, United Kingdom). The quality of the RNA was checked on a bioanalyzer (Agilent Technologies, South Queensferry, United Kingdom). An RNA integrity number (RIN) of >8 proved the integrity of the RNA.

### Microarray hybridization.

Biotinylated fragmented cRNA was hybridized to the Affymetrix chicken genome array (Affymetrix, Santa Clara, CA). This array contains comprehensive coverage of 32,773 transcripts corresponding to >28,000 chicken genes. It also contains 689 probe sets for 684 transcripts from 17 avian viruses, including IBDV. For each experimental group (infected/control in two tissues at three time points in two lines), three biological replicates (three pools of RNA from three birds) were hybridized. Thus, 72 arrays were used in total. Hybridization was performed at 45°C for 16 h in a hybridization oven with constant rotation (60 rpm). The microarrays were then automatically washed and stained with streptavidin-phycoerythrin conjugate (SAPE; Invitrogen) in a Genechip fluidics station (Affymetrix). Fluorescence intensities were scanned with a GeneArray Scanner 3000 (Affymetrix). The scanned images were inspected and analyzed using established quality control measures. Array data have been submitted to Array Express (http://www.ebi.ac.uk/arrayexpress/) under the accession number E-TABM-1129.

### Statistical analysis.

Gene expression data generated from the GeneChip Operating Software (GCOS) were normalized using the PLIER (probe logarithmic intensity error) method (16) within the Affymetrix expression console software package. These normalized data were then analyzed using the limma and FARMS (17) packages within R in Bioconductor (18). Probes with a false discovery rate (FDR) of <0.05 and a fold change of ≥2 were deemed to be significant.

### Analysis of differentially expressed genes.

Gene ontogoly (GO) terms associated with genes differentially expressed (DE) during the host response were analyzed using EasyGO (http://bioinformatics.cau.edu.cn/easygo/). To determine which biological pathways are involved in the responses to viral infection, Pathway Express (http://vortex.cs.wayne.edu/projects.htm) was used. Genes differentially expressed during the host response (FDR < 0.05) were analyzed against a reference background consisting of all genes expressed in the experiment. Factors considered by Pathway Express include the magnitude of a gene's expression change and its position and interactions in any given pathway, thus including an “impact factor” when calculating statistically significant pathways. Anything with a *P* value of <0.25 is deemed significant when using this software.

Genes were clustered by similar expression pattern and analyzed for enriched GO terms and transcription factor binding sites (TFBS) using Expander (v5.2) (http://acgt.cs.tau.ac.il/expander/expander.html). Normalized expression data from control samples were compared to infected samples to examine the host response to IBDV infection. Enrichment analysis of particular GO terms or TFBS within clusters was done using the TANGO and PRIMA functions, respectively, within the Expander package. Use of the Ingenuity Pathway Analysis (IPA) program (Ingenuity Systems) revealed which canonical pathways and physiological functions were affected by IBDV infection in the host (Benjamini-Hochberg multiple testing correction; FDR < 0.05).

### Viral quantification and specific gene expression analysis by quantitative real-time PCR.

TaqMan real-time qRT-PCR was used to quantify viral RNA levels and for confirmation of the microarray results for the mRNA levels of selected genes. Primers (Sigma) and probe (PE Applied Biosystems, Warrington, United Kingdom) (Table 1) were designed using Primer Express (PE Applied Biosystems). Briefly, the assays were performed using 2 μl of total RNA and the TaqMan FAST Universal PCR master mix and one-step RT-PCR Mastermix reagents (PE Applied Biosystems) in a 10-μl reaction. Amplification and detection of specific products were performed using the Applied Biosystems 7500 Fast real-time PCR system with the following cycle profile: one cycle at 48°C for 30 min and 95°C for 20 s, followed by 40 cycles at 95°C for 3 s and 60°C for 30 s. The data are expressed in terms of the cycle threshold (*C_T_*) value, normalized for each sample using the *C_T_* value of 28S rRNA product for the same sample, as described previously (19–21). The final results are shown as 40-*C_T_* using the normalized value, or as fold change from uninfected controls.

**TABLE 1:** Primers used in qRT-PCR analysis of IBDV candidate genes

Primer	Sequence (5′-3′)	Size (bp)	*T_m_* (°C)	%GC
SRFBP1_forward	CACTGCAAGTGAGCGAGCTATT	22	55	50
SRFBP1_reverse	GCAGCTTTAAGTTCAGCAATTTTG	24	52	38
SRFBP1_probe	CAAGACTTGCAACACACCCCCTTCTGA	27	61	52
XRCC3_forward	GCAGACCTGGACACCTTCCA	20	56	60
XRCC3_reverse	CGCACCATGCCTCTTGTG	18	53	61
XRCC3_probe	ACTGCATTACGAAGAGGCTGTCCCTGC	27	63	56
TNFRSF1B_forward	CGAGGAAAGGCTTAAGAAATGTTG	24	54	42
TNFRSF1B_reverse	CGCTGTGACTGCAGCTCTCT	20	56	60
TNFRSF1B_probe	AGCAAATGCCCTCCAGGTCAGCG	23	60	61
CARD9_forward	AACGCAAAGCAGGTGTTCTTC	21	52	48
CARD9_reverse	TCTCCATGAATGCCTCAAAGC	21	52	48
CARD9_probe	ACATTCTACAGCGAACAGGGCGCAA	25	59	52
BLVRA_forward	ACCGTTCATTTTCAGACTGCAA	22	51	41
BLVRA_reverse	CCACTGGTGAAACAGAAATTGATT	24	52	38
BLVRA_probe	CAAGAAGCCTCTCACTTGGATCGAAGAACG	30	63	50
AICDA_forward	GCGCTGGGCCAAAGG	15	50	73
AICDA_reverse	CAACCCATCTTGTTACGCAGGTA	23	55	48
AICDA_probe	ACCTACCTCTGTTATGTTGTGAAGCGCCG	29	63	52
MYBL1_forward	GGCTGCTCAGGAAAAAAAGTATG	23	53	43
MYBL1_reverse	TTCCCTAATGTCTTCCTCCAAGAA	24	54	42
MYBL1_probe	CCCTCTCAAACTTACGTCACAACCACTTGC	30	63	50
TLR2B_forward	GAGCACCAGGGAATGGTTTC	20	54	55
TLR2B_reverse	GCTGTCTGGCTGAGGCTTTT	20	54	55
TLR2B_probe	CACAGAGCCAGCTTCACGTAACCAAAGAA	29	62	48
B6.3_forward	ACTGGAAGTATTTGAGCCGATCA	23	54	43
B6.3_reverse	CGTTGCAAGTTAAGTCTGCAGTTC	24	56	46
B6.3_probe	TGATGCCCGGTGCTTCCCCA	20	58	65
IL13RA2_forward	GCTGCCTGGGCGTCTGT	17	54	71
IL13RA2_reverse	CTCCGATTTGCTCTGGAGATG	21	54	52
IL13RA2_probe	CCACCCAAGTGGAGACCGCTTTCA	24	61	58
CCL19_forward	CGGATAGTGCAGGACTACAGGAT	23	57	52
CCL19_reverse	GAGCCGCTTGCCCTTTG	17	52	65
CCL19_probe	ACATCCCTGCCACCGTGTTCATCA	24	60	54
IFNB_forward	CCTCCAACACCTCTTCAACATG	22	55	50
IFNB_reverse	TGGCGTGTGCGGTCAAT	17	50	59
IFNB_probe	TTAGCAGCCCACACACTCCAAAACACTG	28	61	50
chCCLi3_forward	TGCACCACTTACATAACACACAAGAT	26	55	38
chCCLi3_reverse	TGAAGATGATGGCAGGCTTTG	21	52	48
chCCLi3_probe	CGCGGAACCTCATCCAGAGGCACTA	25	63	60
CXCLi2_forward	GCCCTCCTCCTGGTTTCAG	19	55	63
CXCLi2_reverse	TGGCACCGCAGCTCATT	17	50	59
CXCLi2_probe	TCTTTACCAGCGTCCTACCTTGCGACA	27	61	52
IFNG_forward	GTGAAGAAGGTGAAAGATATCATGGA	26	65	38
IFNG_reverse	GCTTTGCGCTGGATTCTCA	19	51	53
IFNG_probe	TGGCCAAGCTCCCGATGAACGA	22	59	59
IFNA_forward	GACAGCCAACGCCAAAGC	18	63	51
IFNA_reverse	GTCGCTGCTGTCCAAGCATT	20	54	55
IFNA_probe	CTCAACCGGATCCACCGCTACACC	24	63	63
MMP9_forward	CCAGCTACGATGCCGACAA	19	54	58
MMP9_reverse	TCGCTGTTGCCACCATTG	18	50	56
MMP9_probe	CTGCCCCAGCGAGCTGCTCTACA	23	62	65
MX1_forward	TGGACTTCTGCAACGAATTGTC	22	53	45
MX1_reverse	ATCCAGAAGAGTGCTGAAATGTTTG	25	54	40
MX1_probe	TTCACCTCCGCAATCCAGCAAGAGA	25	59	52
IL6_forward	GCTCGCCGGCTTCGA	15	50	73
IL6_reverse	GGTAGGTCTGAAAGGCGAACAG	22	57	55
IL6_probe	AGGAGAAATGCCTGACGAAGCTCTCCA	27	61	52
chCCLi4_forward	CCCTCTCCATCCTCCTGGTT	20	56	60
chCCLi4_reverse	TATCAGCCCCAAACGGAGAT	20	52	50
chCCLi4_probe	CCGCCCTCTTCCCTCAAGCCTC	22	62	68

## RESULTS

### Assessment of IBDV viral load in susceptible and resistant chicken lines.

**FIG 1:**
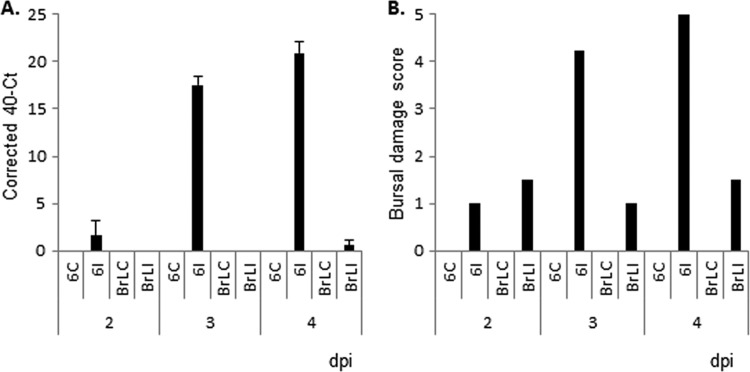
(A and B) Bursal load of IBDV (corrected 40-*C_T_* ± the standard errors) (A) and bursal damage scores (B) at various times postinfection with virulent (F52/70) IBDV. C = control, uninfected; I = infected; 6 = resistant line 6_1_ chickens; BrL = susceptible Brown Leghorn chickens. *n* = 9 birds per group per time point.

**FIG 2:**
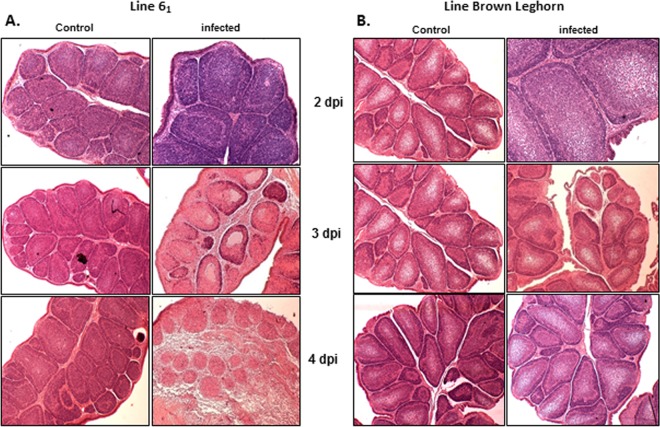
(A and B) Histology staining with hematoxylin and eosin of the bursal damage seen at 2, 3, and 4 dpi in control and infected birds of line 6_1_ (A) and Brown Leghorn (B).

After challenge of the two inbred lines, TaqMan analysis was used to measure viral load in bursal samples from control and infected birds from both lines. It became immediately obvious on analyzing the viral load data that the respective phenotype of the two lines (BrL susceptible and line 6_1_ resistant) had reversed since the lines were last studied in previous decades (13, 14). In repeated experiments, the line 6_1_ birds were susceptible and the BrL birds were highly resistant, with only the odd BrL bird showing detectable viral load in the bursa postchallenge (Fig. 1A). In terms of bursal damage, all control birds had no signs of bursal damage, whereas all infected birds had some bursal damage (Fig. 1B). At 3 and 4 dpi, high bursal viral loads in the line 6_1_ birds correlated with high bursal damage scores, whereas bursal damage scores in infected BrL birds remained low (Fig. 2).

### The viral response upon infection.

As well as representing more than 28,000 chicken genes, the Affymetrix whole-genome array also contains probe sets for avian viral genes, including probes covering the IBD viral genes, i.e., VP1, VP2-3-4, and VP5, thus allowing the simultaneous analysis of changes in viral gene expression postinfection. Probes for each of the viral genes showed an increase in expression in the bursa at days 3 and 4 p.i. in birds from the susceptible line (line 6_1_) but was not seen in birds from the resistant line (BrL), confirming the results shown in Fig. 1. No expression was detected in the spleen from either chicken line (Table 2).

**TABLE 2:** Viral response upon infection

Sample type and Affymetrix ID	Viral gene	Expression data^*a*^																	
		2 dpi						3 dpi						4 dpi					
		Control			Infected			Control			Infected			Control			Infected		
		1	2	3	1	2	3	1	2	3	1	2	3	1	2	3	1	2	3
Bursa																			
Resistant birds																			
NC-004178.CDS1.S1_s_at	Segment A, VP5	18.74	25.09		19.5	22.21	15.65	24.87	18.84	15.91	24.51	18.47	19.45	23.08	13.9	18.49	12.25	17.85	16.99
NC-004178.CDS2.S1_at	Segment A, VP2-3-4	17.06	22.05		18.66	16.74	19.59	13.5	19.62	16.09	15.52	9.01	10.9	21.87	8.7	14.86	17.39	16.77	12.15
NC-004179.CDS1.S1_at	Segment B, VP1	8.66	11.6		4.33	14.55	12.24	9.22	0	5.09	5.69	10.99	1.5	3.33	4.48	9.99	7.18	9.95	7.28
Susceptible birds																			
NC-004178.CDS1.S1_s_at	Segment A, VP5	19.23	19.07	20.11	9.55	36.78		25.43	24.67	28.2	**63.71**	**108.16**	**78.07**	22.76	19.04		**68.55**	**50.93**	**64.95**
NC-004178.CDS2.S1_at	Segment A, VP2-3-4	15.51	22.04	19.22	15.39	5.95		31.69	29.1	18.29	**56.2**	**72.77**	**68.43**	18.18	17.26		**52.09**	**32.28**	**47.9**
NC-004179.CDS1.S1_at	Segment B, VP1	5.95	5.55	6.1	5.12	11.04		7.05	4.32	3.36	**17.49**	**26.64**	**26.25**	4.34	5.87		**14.96**	5.07	**13.3**
Spleen																			
Resistant birds																			
NC-004178.CDS1.S1_s_at	Segment A, VP5	18.71	21.86	22.9	19.1	25.23		24.97	17.98	15.31	26.13	20.16		20.18	16.88	26.93	22.88	21.6	20.54
NC-004178.CDS2.S1_at	Segment A, VP2-3-4	16.11	25.56	27.96	9.89	23.67		26.44	20.82	27.2	21.85	27.13		26.95	20.57	25.71	21.56	22.97	23.91
NC-004179.CDS1.S1_at	Segment B, VP1	4.55	1.14	0	3.05	0.01		3.01	0.01	0	1.8	5.11		0.69	0.98	0	2.93	0.4	0.96
Susceptible birds																			
NC-004178.CDS1.S1_s_at	Segment A, VP5	19.62	24.25	24.81	22.11	23.68	18.27	24.89	20.6	19.16	20.15	23.44	31.8	29.72	24.68	20.9	23.82	21.67	18.98
NC-004178.CDS2.S1_at	Segment A, VP2-3-4	17.66	6.83	17.66	19.8	20.62	18.75	21.63	19.46	21.29	29.75	18.58	25.87	18.17	27.85	19.54	27.21	18.84	18.51
NC-004179.CDS1.S1_at	Segment B, VP1	0.75	4.09	3.96	0	0	3.55	2.31	5.9	0	0.17	2.77	0	0.98	0	3.59	1.55	0	0

a Significant values are indicated in boldface.

### Host response to IBDV infection.

After infection with virus, no genes were seen to be significantly differentially expressed in the resistant BrL birds. Gene expression differences between infected and control birds of the susceptible line 6_1_ at 2, 3, and 4 dpi were therefore analyzed, with a view to examining the host innate immune response to infection by IBDV. At day 2 there was an initial response in the spleen (121 DE genes), although no gene expression changes were noted in the bursa. It is not until day 3 that large gene expression changes were noted (1,228 DE genes in the spleen and 3,069 DE genes in the bursa), which continued at day 4 (826 DE genes in the spleen and 3,268 DE genes in the bursa). A larger response was seen in the bursa than in the spleen, which was expected, since the bursa is the main target organ for the disease. Genes upregulated during the host response included genes, e.g., *IL-6*, *IL-8*, *MX1*, *IFIT5*, *TLR3*, and *NOS2A*, that had already been identified as being upregulated after IBDV infection in previous studies. Many other genes with known immune function were involved in the response to IBDV, such as the matrix metalloproteins *MMP9*, *MMP7*, *MMP1*, and *MMP3*, the chemokines *CCLi7*, *CCLi4*, *CCL5*, and *CCL19*, the interferon (IFN)-induced transmembrane proteins *IFITM1*, *IFITM3*, and *IFITM5*, as well as *GBP*, *ART1*, *PDIP1*, *OASL*, *CSF3*, *IRF10*, and *TLR15*, to name but a few. Genes downregulated during the early host response included *BLVRA*, *MYBL1*, *AICDA*, *SFRP1*, *VPREB3*, *SFTPA2*, and *TLR7*. For a full list of the genes involved in both bursa (3,658 DE probes) and spleen (1,476 DE probes) see Table S1 in the supplemental material.

In order to confirm which biological processes were involved during infection of the birds and to determine whether there was any that had not been identified in previous, more-focused studies, we decided to analyze the gene ontology (GO) functional annotations of the genes being differentially expressed. The microarray data produced for the host response (infected versus control susceptible birds) in the spleen were analyzed with EasyGO (22), which examines DE genes for their association with particular GO terms compared to the microarray background as a whole. The response in the spleen was analyzed as opposed to that in the bursa since the large degree of cell damage occurring in the bursa would substantially mask the immune response. The genes differentially expressed during this experiment were seen to be involved in processes such as “immune response,” “apoptosis,” “response to stimulus,” and “cytokine production,” which is what would be expected in this type of infection.

**FIG 3:**
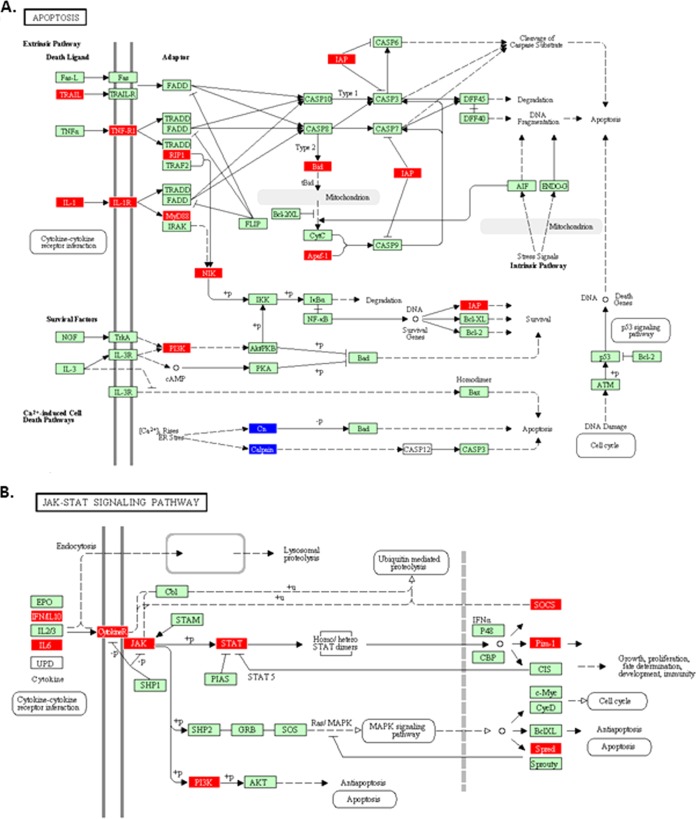
Pathway Express analysis of the host response to IBDV infection in the spleen. Many genes in the apoptosis (A) and TLR pathways (B) are upregulated (red). Genes in blue are downregulated.

**FIG 4:**
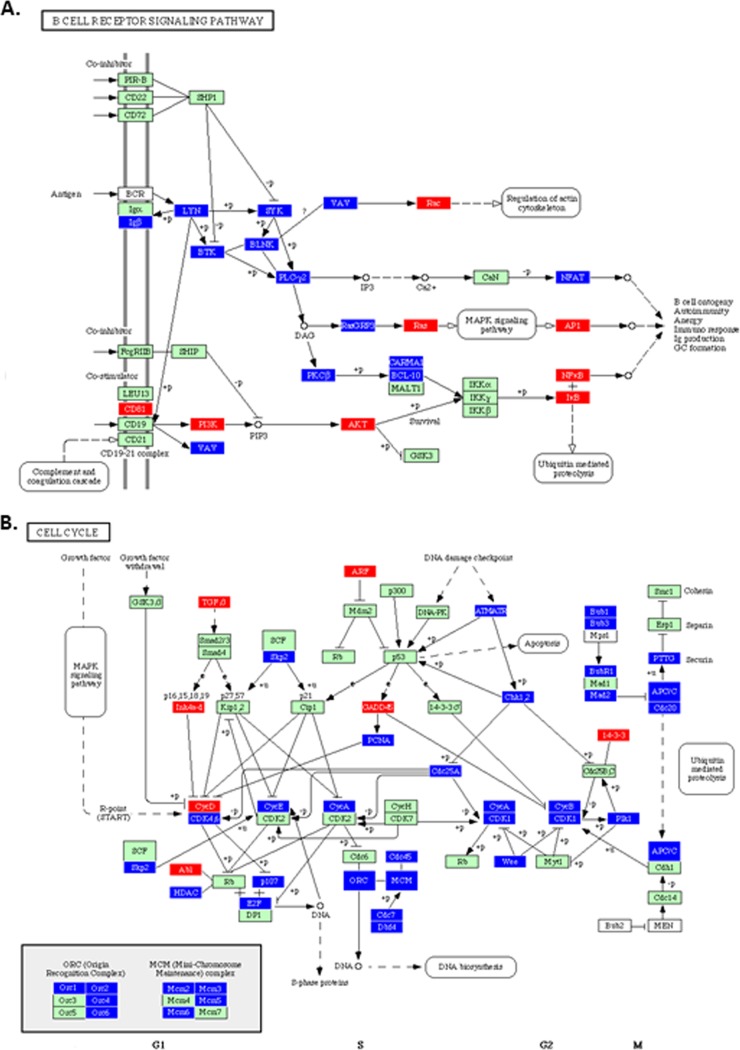
Pathway Express analysis of the host response to IBDV infection in the bursa. Many genes in the B cell receptor signaling pathway (A) are downregulated (blue), as are many genes involved in the cell cycle (B).

The data were also analyzed using Pathway Express (23), which, based upon the KEGG pathways (24), pictorially illustrates the genes that are up- or downregulated in any given biological pathway. Figure 3 shows examples using the data from the spleen at 4 dpi. Genes involved in the extrinsic apoptosis pathway and the Toll-like receptor signaling pathway, which play integral roles during the innate immune response, are upregulated. In the bursa, genes involved in the B-cell receptor signaling and cell cycle pathways were dramatically downregulated (Fig. 4). Many of the biological responses seen in the bursa will be due to viral replication and cell damage and will not be antiviral responses *per se*. It must be borne in mind that these diagrams are based on the human pathways and so in some cases are not completely demonstrative of the chicken pathways, i.e., avian-specific genes are not represented. Other pathways seen to be significantly (FDR-corrected *P* value of <0.25) involved are shown in Table 3.

**TABLE 3:** Pathway Express analysis of the host response to IBDV infection in the spleen

Rank	Pathway	Impact factor	Input genes/genes in pathway	Corrected gamma *P* value
1	Leukocyte transendothelial migration	41.149	12/119	5.68E–17
2	Cell adhesion molecules (CAMs)	30.238	14/134	2.30E–12
3	Phosphatidylinositol signaling system	23.129	1/76	2.18E–09
4	Cytokine-cytokine receptor interaction	17.66	39/263	3.99E–07
5	Circadian rhythm	12.371	1/13	5.67E–05
6	Jak/STAT signaling pathway	9.513	23/155	7.77E–04
7	Toll-like receptor signaling pathway	7.164	17/102	0.006318549
8	Graft-versus-host disease	6.712	4/42	0.00937956
9	Regulation of autophagy	6.442	1/35	0.011856721
10	Proteasome	6.319	1/48	0.013186967
11	TGF-β signaling pathway	6.093	5/87	0.016020412
12	Complement and coagulation cascades	6.064	10/69	0.016424379
13	Neuroactive ligand-receptor interaction	4.986	14/256	0.040901989
14	Antigen processing and presentation	4.647	7/89	0.054156592
15	Axon guidance	4.491	14/129	0.061550973
16	Type I diabetes mellitus	4.456	3/44	0.063337096
17	Systemic lupus erythematosus	4.425	8/144	0.064960096
18	Allograft rejection	4.361	3/38	0.068436465
19	Epithelial cell signaling in Helicobacter pylori infection	4.348	12/68	0.069163823
20	Hematopoietic cell lineage	4.294	13/87	0.072264239
21	Type II diabetes mellitus	4.231	5/45	0.076047469
22	Apoptosis	4.198	13/89	0.078103058
23	Natural killer cell mediated cytotoxicity	4.168	10/135	0.080017155
24	Basal cell carcinoma	3.776	2/55	0.109438052
25	ABC transporters	3.54	7/44	0.131720505
26	Adipocytokine signaling pathway	3.205	7/67	0.170550181
27	Adherens junction	3.05	4/78	0.191803644
28	Renal cell carcinoma	2.845	10/69	0.223526256
29	Mitogen-activated protein signaling pathway	2.758	22/272	0.238326642
30	Long-term depression	2.695	6/75	0.249569101

In order to cluster the genes demonstrated to be involved in the host response to IBDV into groups with similar expression profiles, the CLICK algorithm within the Expander program (25) was used. The upregulated genes formed four distinct clusters, while the downregulated genes all cluster together in a fifth group.

**FIG 5:**
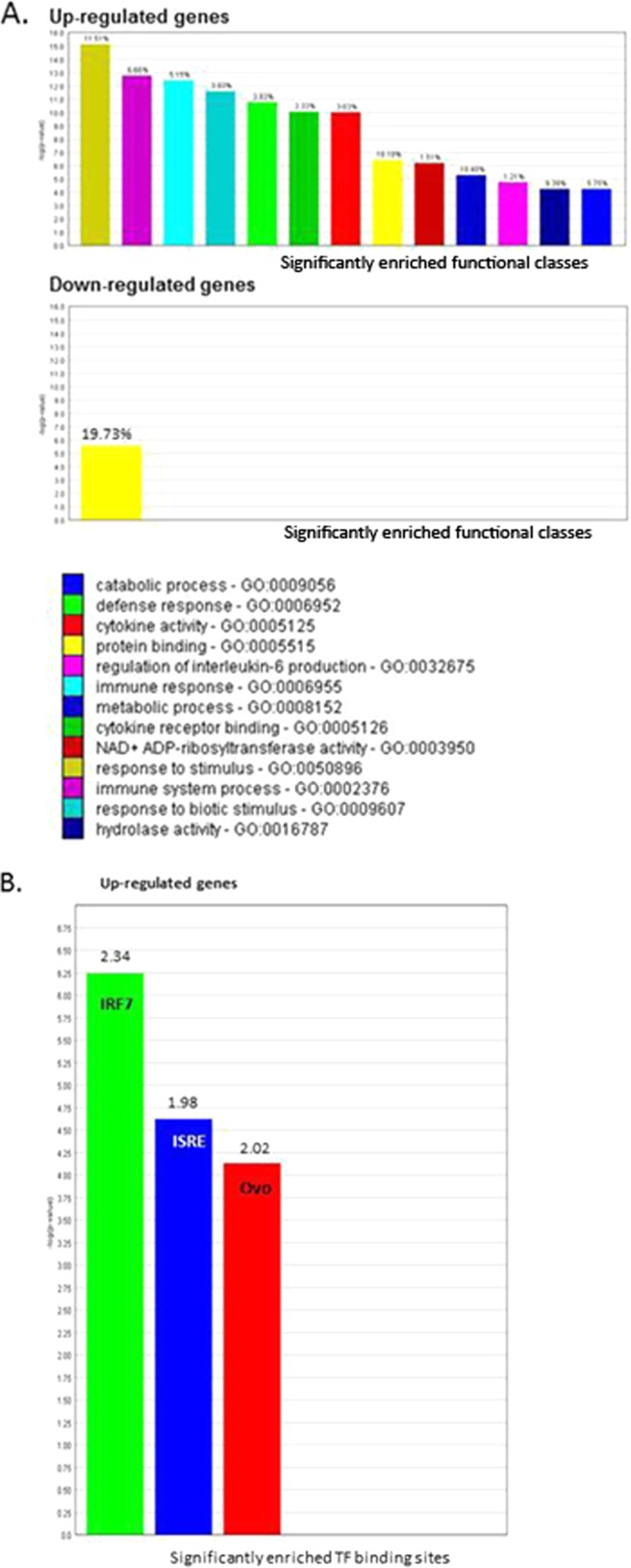
Over-representation analysis using the Expander program. (A) The GO biological processes which are significantly enriched during the host response to IBDV infection. The frequency of genes of a functional class within the examined set is described as a percentage of the total. (B). Transcription factor binding sites present in differentially expressed genes that are significantly overrepresented in upregulated genes during the host response to IBDV infection. The frequency ratio (frequency of the set divided by the frequency of the background) is shown.

Expander was then also used to look for enrichment of GO terms associated with genes, genomic locations and TFBS within the clustered DE genes (834 upregulated and 377 downregulated). The upregulated genes were over-represented in biological processes such as “immune response,” “response to stimulus,” “cytokine activity,” and other functions associated with viral infection. Only “protein binding” was highlighted among the downregulated genes. Examination of the microarray data did not show any enrichment for a particular genomic location among the DE genes. Upregulated genes showed an enrichment of TFBS for ISRE (IFN stimulatory response element) and IRF7 (interferon regulatory factor 7). IRF7 regulates transcription of type I IFN genes and IFN-stimulated genes by binding to ISRE motifs in their promoters. Genes containing a binding site for the Drosophila transcription factor Ovo (presumably the chicken orthologue thereof) was also over-represented. No TFBS were seen to be enriched within the downregulated array genes (Fig. 5).

**FIG 6:**
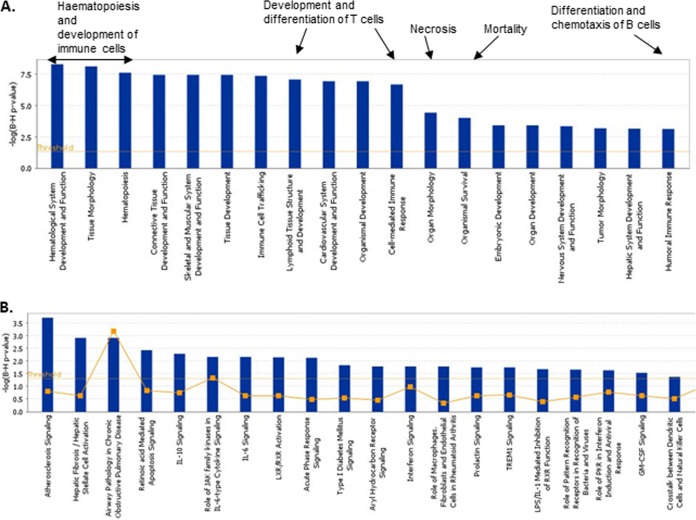
IPA results for the host response to IBDV infection. (A) The most highly represented (*P* < 0.05) physiological functions as revealed after IPA was used to evaluate genes differentially expressed during the host response to IBDV (in the spleen). Specific functions within groups are highlighted. (B) The most highly represented canonical pathways as revealed after IPA was used to evaluate genes differentially expressed during the host response to IBDV (in the spleen). The line represents the ratio of genes represented within each pathway.

Complementing the findings from Pathway Express, IPA was also used to identify the physiological functions and biological pathways most highly involved during the host response to IBDV infection. The most significant physiological functions are represented in Fig. 6A. It can be clearly seen that processes involving B and T cell development and differentiation constitute many of the pathways highlighted. Tissue development, necrosis, and mortality all seem to play an important role during IBDV infection. Pathways affected by IBDV infection are shown in Fig. 6B. Among the upregulated genes, those involved in signaling from several cytokine receptors (*IFN*, *IL-6*, *IL-10*, and *GM-CSF*/*CSF-2*), as well as in apoptosis, were differentially expressed. Genes involved in the activation of hepatic stellate cells were also differentially expressed. Interestingly, Ma et al. (26) recently described the effects of IBDV on Kupffer cells, macrophages found in the lining of the liver, suggesting that the liver is a site of IBDV infection and therefore possibly the innate immune response. Analysis of the downregulated genes highlights genes that are involved in endothelial cell development, proliferation, and migration.

### Differences between susceptible and resistant lines.

Resistance to IBD between the two lines could be due to a number of mechanisms. For example, BrL birds could simply express certain genes, such as those involved in key innate immune responses, at a constitutively higher level than line 6_1_ birds, and thus mount a stronger innate response upon infection, limiting viral replication and thus disease. Alternatively, after infection, BrL birds could upregulate the expression of key immune function genes to a greater degree than line 6_1_ birds, thus mounting a stronger induced immune response. Either or both mechanisms could contribute to IBD resistance.

There were genes that showed large expression differences between the two lines, even before infection, which in turn led to the differing responses seen upon infection. Examination of the control birds in the two lines showed considerable differences in expression levels of certain genes, including *BLB1*, *SRFBP1*, *XRCC3*, *TNFRSF1B*, *GNG4*, and *IFITM3*, all of which were more highly expressed in the resistant (BrL) than in the susceptible line (line 6_1_). Some of these genes may therefore play an important role in disease resistance (382 DE probes in the spleen and 160 DE probes in the bursa: see Table S2 in the supplemental material).

Upon infection, differences in gene expression were also seen between the two lines. Genes more highly expressed in the resistant BrL line included *BLB1*, *CARD9*, *BLVRA*, *AICDA*, *MYBL1*, *SFRP1*, *B6.3*, *VPREB3* and *MMP13*, whereas genes more highly expressed in the susceptible line included *MMP9*, *IFNA*, *CCL5*, *MMP7*, *AVD*, *NOS2A*, *CXCLi2*, *IL-6*, *LYG2*, *GBP*, *CCL19*, *IFITM1*, *IFITM3*, and *IFITM5*. Of course, “more highly expressed” could also mean “less downregulated.” Again, it seems reasonable to assume that some of these genes may play an important role in resistance/susceptibility to IBDV. A full list of genes differentially expressed between the two lines in both the bursa (3,659 DE probes) and the spleen (1,570 DE probes) is given in Table S3 in the supplemental material.

### Verification of the microarray results.

**FIG 7:**
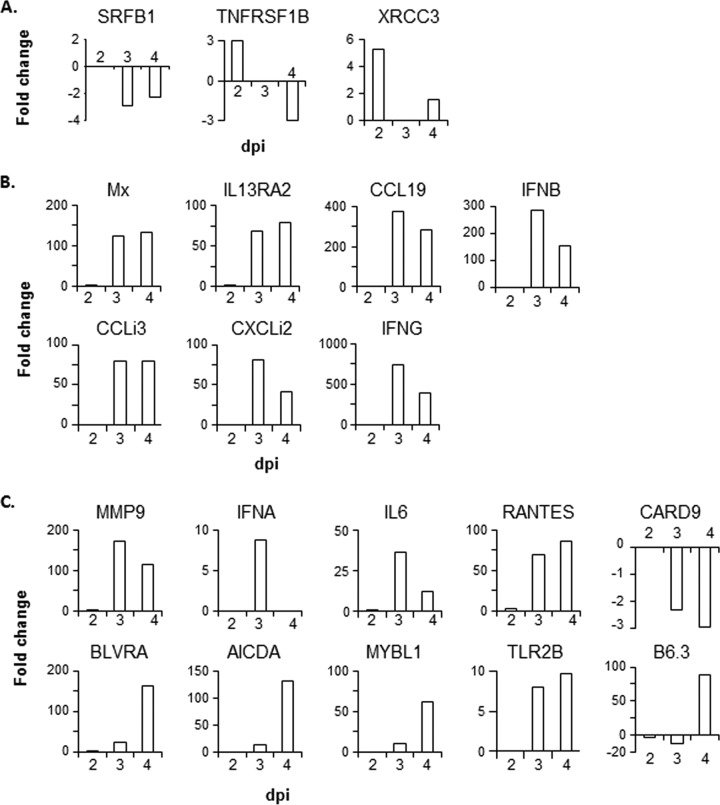
qRT-PCR analysis of 20 genes differentially expressed during IBDV infection. (A) Representative genes with inherent differences in expression between susceptible and resistant control birds. (B) Representative genes with expression changes during the host response in the susceptible line. (C) Representative genes with differential gene expression between the susceptible and resistant lines during the host response.

Twenty-one genes were chosen for verification by qRT-PCR, including genes involved in the host response and genes differentially expressed between the susceptible and resistant lines (either inherently or during the course of infection) (Table 4). Of the 20 genes tested, differential expression in the microarray was confirmed in the qRT-PCR analysis for 17. However, the microarray results for *SRFBP1*, *TNFRSF1B*, and *CARD9* were not replicated in the qRT-PCR analysis (note that the qRT-PCR assay did not work for *BLB1*) (Fig. 7).

**TABLE 4:** Genes chosen for confirmation by qRT-PCR

Gene^*a*^	GenBank accession no.	Description	Fold change^*b*^
**BLB1**	NM_001044694	MHC class II antigen B-F minor heavy chain	203–263*
**SRFBP1**	XM_424408	Serum response factor binding protein 1	24–25*
TNFRSF1B	NM_204439	Tumor necrosis factor receptor superfamily, member 1B	8–9*
**XRCC3**	NM_001006489	X-ray repair complementing defective repair in Chinese hamster cells 3	14–20*
**AICDA**	XM_416483	Activation-induced cytidine deaminase	8–57†
**B6.3**	X92865	B cell marker chB6 (Bu-1)	6–42†
**BLVRA**	XM_418872	Biliverdin reductase A	8–42†
CARD9	XM_425329	Caspase recruitment domain family, member 9	3–16†
**MYBL1**	NM_205232	Myeloblastosis oncogene-like 1	7–37†
**TLR2B**	AB046533	Toll-like receptor 2, type 2	4–5†
**CCL19**	XM_424980	CCL19 homeostatic chemokine	84–99‡
CCLi3	NM_204720	CCLi3 MIP family chemokine	76–100‡
**CCLi4**	NM_001045832.1	CCL5 MIP family chemokine (RANTES)	209–318‡
**CXCLi2**	NM_205498	CXCLi2 (IL-8 homologue)	50–65‡
IFNA	EU334503	IFN-α	470–1,359‡
**IFNB**	NM_001024836	IFN-β	51–85‡
**IFNG**	NM_205149	IFN-γ	32–72‡
**IL13RA2**	NM_001048078	IL-13 receptor-α2	53–111‡
IL-6	NM_204628	IL-6	52–198‡
MMP9	NM_204667	Matrix metalloprotein 9	1,099–1,782‡
Mx1	NM_204609	Myxovirus resistance gene 1	60–69‡

a Genes indicated in boldface are potential candidate resistance genes.

b *, Inherently higher expression in the resistant line; †, higher expression in response to infection in the resistant than in the susceptible line; ‡, upregulated in response to infection in the susceptible line.

### Potential candidate genes for IBDV resistance.

In contrast to other avian pathogens, very little information is available regarding potential quantitative trait loci (QTL) regions for resistance to IBDV. The only published material is to be found in a Ph.D. thesis (14) that suggests that several genomic regions are potentially involved in resistance phenotypes, defined by death, bursal damage, IBDV genomic RNA levels, and IFN-γ mRNA levels.

Due to the lack of robust accompanying genetic data, we had to identify potential candidate genes for resistance from our expression data alone. We found genes that are differentially expressed between susceptible and resistant lines either inherently or in response to IBDV infection. Examination of these genes, along with their known biology and how they might play a role in the pathways and biological networks identified in our analyses, allowed us to identify potential candidate genes for susceptibility/resistance to IBDV infection. Table 4 lists candidate genes (indicated in boldface) for resistance to IBDV, as determined by their differential expression between susceptible and resistant lines.

## DISCUSSION

IBDV preferentially replicates in IgM^+^ B cells, which it enters after binding to cellular receptors, some of which have been identified, and induces host cell apoptosis, with both VP2 and VP5 playing a role in both binding and apoptosis. VP2 forms a subviral particle which binds to HSP90 (27). Other host molecules, including p53 binding protein (TP53BP1), stathmin (STMN1), and chondroitin sulfate, are also targets for VP2 (28). VP5 interacts with the voltage-dependent anion channel 2 (VDAC2) protein (29) and with the p85α regulatory subunit of phosphatidylinositol 3-kinase (PI3K) (30). Integrin α4β1 is also a specific host binding receptor for IBDV (31). Comparing gene expression levels between control birds of each line, we found higher mRNA expression levels of some of these receptor genes (*HSPCB* [*HSP90B*], *TP53BP1*, *STMN1*, and *ITGB1*) in the resistant line (BrL) than in the susceptible line (6_1_). It seems counterintuitive that the resistant line should have more receptors for the virus than the susceptible line. This presumably reflects some mechanism whereby viral entry into the cell is limited. The Jun NH_2_-terminal kinase and p38 mitogen-activated kinase pathways (30, 32) have previously been suggested to be involved in the pathogenesis of IBD. We confirm here the involvement of these pathways during IBDV infection and highlight the whole spectrum of genes in these pathways whose expression is altered. The importance of apoptosis in IBD pathogenesis is clear from the EasyGO analysis and Pathway Express highlights the affected molecules, also showing upregulation of PI3K. IBDV activates PI3K/Akt signaling through binding of the nonstructural VP5 protein to the p85α regulatory subunit of PI3K, resulting in the suppression of premature apoptosis and improved virus growth after infection (30). In the bursa, there is also considerable downregulation of genes involved in the B cell receptor signaling pathway and in the cell cycle. The former probably reflects the huge reduction in B cell numbers as they are destroyed by the infection. It remains to be seen whether VP5, or indeed any of the other IBDV proteins, also targets the cell cycle.

Generally, T cells are refractory to IBDV infection with IBDV but promote virus clearance, by mechanisms that are not well understood. Rauf et al. (33) showed that CD4^+^ and CD8^+^ T cells enter the infected bursa and that cytotoxic T cells play a role in clearing infected cells through the perforin-granzyme pathway, as identified by the upregulation of mRNA expression levels for perforin and granzyme, DNA repair and apoptotic proteins, high-mobility protein group and poly(ADP-ribose) polymerase (PARP) in the bursa, and the presence of perforin- and granzyme-expressing CD8^+^ T cells. Similarly in the present study, we observed upregulation of granzyme A, various PARP genes, and also IFN-γ. Rauf et al. (33) also noted a decrease in NK-lysin mRNA expression levels, suggesting a reduced role for NK cells. In contrast, the present study demonstrated a 5-fold upregulation of NK lysin in the bursa at 3 dpi, rising to 15-fold at 4 dpi, suggesting that in these birds both cytotoxic T cells and NK cells are involved in the response to the virus.

The chicken has a somewhat different repertoire of pattern recognition receptors (PRR), including Toll-like receptors (TLR), to that of mammals (34), but the repertoire of potential pathogen-associated molecular patterns (PAMP) recognized by these PRR is thought to be similar. Little is understood of the effects infection with different classes of pathogens have on the expression repertoire of the different TLR. In the present study, during the early stages of IBDV infection in the bursa, mRNA expression levels of *TLR1LB*, *TLR2A*, *TLR3*, *TLR4*, and *TLR15* (and *TLR21* in the spleen) and the cytosolic PRR *MDA5* were upregulated, whereas those of *TLR1LA*, *TLR2B*, and *TLR7* were downregulated. Other studies have also reported this differential regulation of *TLR3* and *TLR7* following infection with a classical strain of IBDV (33, 35). TLR3 recognizes viral dsRNA, whereas TLR7 recognizes viral single-stranded RNA; the genome of IBDV is dsRNA, and upregulation of TLR3 might represent a positive feedback loop to drive the innate inflammatory antiviral response. MDA5 also recognizes viral dsRNA, and its mRNA expression levels were also upregulated (70-fold at 3 dpi compared to 9-fold for TLR3) in response to IBDV infection.

It is more difficult to explain the differential mRNA expression patterns of some of the TLRs that are thought to generally recognize components of the surface of pathogens, particularly bacteria. The precise ligand of the chicken-unique TLR15 remains to be determined. It has been described to recognize a variety of PAMP, including diacylated lipopeptides (36) and a yeast-derived agonist (37). We previously reported increased expression of *TLR15* after infection with Marek's disease virus (38). Together with the data presented in the present study, this implies a role for TLR15 in antiviral responses and that it perhaps recognizes a surface component of viruses. It is interesting to note the differential response of the *TLR1* and *TLR2* paralogues, i.e., *TLR1LB* and *TLR2A* mRNA expression levels are upregulated, and those of *TLR2B* and *TLR1LA* are downregulated. Moreover, TLR2-1 and TLR1-2 heterodimers cooperatively signal the presence of PGN, diacylated lipopeptides and MALP-2, whereas TLR2-1 and TLR1-1 heterodimers did not recognize MALP-2. Both combinations, however, recognized the triacylated lipopeptide, Pam3 (39). It is therefore clear that the two different heterodimer combinations discriminate between different ligands and that they might differentially recognize surface components of viruses.

Analysis of the samples from IBD-susceptible and -resistant birds, with or without infection with IBDV, using whole-genome microarrays identified genes that either show different inherent levels of gene expression (without infection) between the lines or that are transcribed differently after infection, thus potentially eliciting differing host responses. They are thus prime candidates for future testing in genetic mapping studies, either as targets for knockout experiments or as targets for direct interaction with IBDV proteins.

## Supplementary Material

Supplemental material
