# Supplementary material for: Analysis of the Early Immune Response to Infection by Infectious Bursal Disease Virus in Chickens Differing in Their Resistance to the Disease
Source: J Virol. 2014 Dec 10;89(5):2469–82. doi: 10.1128/JVI.02828-14 (PMC4325706; doi:10.1128/JVI.02828-14)
Supplement: Supplemental material [file supp_89_5_2469__index.html]

Analysis of the Early Immune Response to Infection by Infectious Bursal Disease Virus in Chickens Differing in Their Resistance to the Disease — Supplemental material 

# Analysis of the Early Immune Response to Infection by Infectious Bursal Disease Virus in Chickens Differing in Their Resistance to the Disease

## Supplemental material

**Files in this Data Supplement:**

- Supplemental file 1 -

  Table S1 (Host response to infection with IBDV in the bursa and in the spleen.)

  XLS, 982K
- Supplemental file 2 -

  Table S2 (Gene expression differences found to be inherent between susceptible and resistant lines in the bursa and in the spleen.)

  XLS, 144K
- Supplemental file 3 -

  Table S3 (Genes found to be differentially expressed between susceptible and resistant lines in response to IBDV infection in the bursa and in the spleen.)

  XLS, 980K
